# Rapid and simple detection of foot‐and‐mouth disease virus: Evaluation of a cartridge‐based molecular detection system for use in basic laboratories

**DOI:** 10.1111/tbed.12744

**Published:** 2017-11-09

**Authors:** K. V. Goller, V. Dill, M. Madi, P. Martin, Y. Van der Stede, V. Vandenberge, B. Haas, S. Van Borm, F. Koenen, C. J. Kasanga, N. Ndusilo, M. Beer, L. Liu, V. Mioulet, B. Armson, D. P. King, V. L. Fowler

**Affiliations:** ^1^ Institute of Diagnostic Virology Friedrich‐Loeffler‐Institut Greifswald ‐ Insel Riems Germany; ^2^ Vesicular Disease Reference Laboratory The Pirbright Institute Pirbright Surrey UK; ^3^ Enigma Diagnostics Limited Porton Down Salisbury UK; ^4^ Unit of Coordination of Veterinary Diagnosis, Epidemiology and Risk Analysis Operational Directorate of Interactions and Surveillance Veterinary and Agrochemical Research Centre Brussels Belgium; ^5^ Sokoine University of Agriculture Morogoro Tanzania; ^6^ National Veterinary Institute Uppsala Sweden

**Keywords:** cartridge‐based real‐time RT‐PCR, disease control, foot‐and‐mouth disease virus, rapid diagnostics

## Abstract

Highly contagious transboundary animal diseases such as foot‐and‐mouth disease (FMD) are major threats to the productivity of farm animals. To limit the impact of outbreaks and to take efficient steps towards a timely control and eradication of the disease, rapid and reliable diagnostic systems are of utmost importance. Confirmatory diagnostic assays are typically performed by experienced operators in specialized laboratories, and access to this capability is often limited in the developing countries with the highest disease burden. Advances in molecular technologies allow implementation of modern and reliable techniques for quick and simple pathogen detection either in basic laboratories or even at the pen‐side. Here, we report on a study to evaluate a fully automated cartridge‐based real‐time RT‐PCR diagnostic system (Enigma MiniLab^®^) for the detection of FMD virus (FMDV). The modular system integrates both nucleic acid extraction and downstream real‐time RT‐PCR (rRT‐PCR). The analytical sensitivity of this assay was determined using serially diluted culture grown FMDV, and the performance of the assay was evaluated using a selected range of FMDV positive and negative clinical samples of bovine, porcine and ovine origin. The robustness of the assay was evaluated in an international inter‐laboratory proficiency test and by deployment into an African laboratory. It was demonstrated that the system is easy to use and can detect FMDV with high sensitivity and specificity, roughly on par with standard laboratory methods. This cartridge‐based automated real‐time RT‐PCR system for the detection of FMDV represents a reliable and easy to use diagnostic tool for the early and rapid disease detection of acutely infected animals even in remote areas. This type of system could be easily deployed for routine surveillance within endemic regions such as Africa or could alternatively be used in the developed world.

## 1 INTRODUCTION

Foot‐and‐mouth disease (FMD) is a globally important, highly contagious, transboundary disease of domestic and wild cloven‐hoofed animals. The disease is of great economic importance due to its ability to rapidly spread in susceptible livestock populations causing outbreaks of immense impact on animal health, welfare, productivity and trade (Rodriguez & Gay, 2011). The causative agent FMD virus (FMDV) is a member of the genus *Aphthovirus* within the family *Picornaviridae* that has a single‐stranded positive‐sense RNA genome of approximately 8.4 kb (Jamal & Belsham, 2013), which exists as seven serotypes (O, A, C, Asia‐1 and Southern African Territories [SAT] 1, 2 and 3). The ability to control FMD is strongly correlated to economic status, with high‐income countries having eradicated the disease (e.g., Europe and North America), middle‐income countries on the way to doing so (e.g., South America and Southeast Asia), and FMD remaining largely uncontrolled in low‐income regions (e.g., sub‐Saharan Africa) (Knight‐Jones & Rushton, 2013). The presence of FMD in endemic countries poses a major risk for introduction of disease into FMD‐free countries (Grubman & Baxt, 2004). Therefore, it is of utmost importance to detect the disease at an early stage to limit the impact of outbreaks and to take rational steps towards timely detection and eradication (Grubman & Baxt, 2004).

Effective control of FMD usually relies upon rapid field diagnosis, accurate laboratory detection and confirmation which are undertaken at designated reference laboratories using laboratory‐based diagnostic assays recommended by the World Organization for Animal Health (OIE) (Callahan et al., 2002; Ferris et al., 2009). However, this pipeline requires that samples are transported from farm to laboratory for analysis, which can delay critical decision‐making. Furthermore, reference laboratory infrastructure is mainly focussed in developed countries like those in Europe or Northern America. As a result, efforts have been made to develop portable field assays (so‐called point‐of‐care or pen‐side assays) for FMDV detection. Antigen detection has been incorporated into immunochromatographic lateral‐flow devices (Ag‐LFD) (Ferris et al., 2009); however despite these tests being highly portable, rapid and simple to use, they often have a low analytical sensitivity. Furthermore, new portable molecular platforms have also been developed for field‐based real‐time RT‐PCR (rRT‐qPCR) (King et al., 2008) which integrate nucleic acid extraction, thermal cycling and calling of results without user intervention and are appropriate for use by non‐specialists (Madi et al., 2012). These rRT‐qPCR platforms such as the Enigma Field Laboratory^®^ (FL), which utilize lyophilized reagents, have been successfully used within endemic settings for the rapid, simple, on‐farm detection of FMDV in a range of clinical samples (Howson et al., 2015). These proof‐of‐concept studies provide evidence to support the use of highly sensitive molecular assays in field settings; however, there may be other scenarios where it would be beneficial to analyse the samples at higher throughput near farms such as local laboratories using similar fully automated platforms. In this study, we report the performance of a simple‐to‐use cartridge‐based automated rRT‐qPCR platform, the Enigma MiniLab^®^ (ML), onto which the gold‐standard OIE‐recommended rRT‐qPCR assay for detection of FMDV was transferred and evaluated for its analytical sensitivity and robustness in European and African laboratories. In contrast to the FL that can be used on a farm, the ML is designed for use in basic laboratories and is a modular system that could harbour up to six units for independent detection and reporting of FMDV‐positive samples.

## 2 MATERIALS AND METHODS

### 2.1 The Enigma ML^®^ system

The cartridge‐based Enigma ML^®^ system combines fully automated nucleic acid extraction with rRT‐qPCR and autonomous result calling. The system consists of one control module and up to six processing modules. Each processing module can independently run one sample, with a maximum of six samples running simultaneously. The cartridges can be stored at room temperature, are single‐use and fully contained. They include tools and reagents (buffers and magnetic beads) for the robotic nucleic acid extraction, freeze‐dried rRT‐qPCR mixes and a polymer capillary for thermal cycling and fluorescence detection. The only user interaction required is loading 2 ml of sample and starting the run with the touch screen interface. The time from sample loading to reporting of results is less than 90 min.

As previously described for the Enigma FL^®^ prototype for on‐farm use (Howson et al., 2015), the FMDV assay on the Enigma ML^®^ platform is a lyophilized form of the OIE‐recommended “gold standard” rRT‐qPCR that targets the conserved 3D region of the FMDV genome (Callahan et al., 2002). The cartridge also includes an internal control that is run together with the FMDV assay. To this means, MS2 phage RNA is added to the sample before the nucleic acid extraction and is subsequently detected along with the FMDV target in a duplex rRT‐qPCR (assay modified from (Rolfe et al., 2007)).

### 2.2 Comparison with standard laboratory methods

Two European laboratories (TPI: The Pirbright Institute, Pirbright, United Kingdom and FLI: Friedrich‐Loeffler‐Institut, Greifswald, Germany) and one African laboratory (SUA: Sokoine University of Agriculture, Morogoro, Tanzania) tested samples in parallel with the Engima ML^®^ FMDV cartridge and with the same assay run on standard laboratory equipment. At TPI, the laboratory work was undertaken in June 2015, at FLI between March and April 2015 and the studies at SUA were done undertaken in May 2015.

At TPI, all standard tests, with the exception of milk samples, were performed on nucleic acid extracted using the MagNA Pure LC Total Nucleic Acid Isolation Kit (Roche) and the MagNA Pure LC automated platform as per manufacturer's guidelines (500 μl:200 μl of sample and 300 μl of lysis/binding buffer). For milk, RNA was extracted using the MagMAX™‐96 Viral RNA Isolation Kit (Life Technologies) on a MagMAX™ Express 96 Extraction Robot, as per manufacturer's guidelines.

At FLI and SUA, all reactions were performed on nucleic acid manually extracted using QIAamp Viral RNA Mini Kit (Qiagen, Hilden, Germany) and TRIzol^®^ reagent (Invitrogen), respectively. Samples were assayed in duplicate on bench‐top real‐time PCR machines (FLI and TPI: Stratagene Mx3005P, Agilent Technologies, Stockport, UK; SUA: 7500 Fast Real‐Time PCR System, Applied Biosystems, Foster City, USA). Reagents, parameters and thermal cycling were as reported by Shaw et al. (2007). All samples were tested in duplicate.

### 2.3 Analytical sensitivity of the Enigma ML^®^

The analytical sensitivity of the Enigma ML^®^ was established at TPI using a 10‐fold dilution series of a 10% (w/v) suspension of bovine epithelium in M25 phosphate buffer (35 mM Na_2_HPO_4_, 5.7 mM KH_2_PO_4_, pH 7.6) that was spiked with cell culture supernatant of FMDV O/UAE/02/2003. For the test, RNA from the dilution series was extracted with either the Enigma ML^®^ or the MagNA Pure LC robot. RNA extracted with the Enigma ML^®^ was then tested with either the standard laboratory method on an Mx3005P or with the Enigma ML^®^; RNA extracted with the MagNA Pure LC was only tested on the Mx3000P.

### 2.4 Diagnostic performance of the Enigma ML^®^ in Europe and Africa

To evaluate the diagnostic performance of the Enigma ML^®^ with clinical specimens, archival samples from animal experiments (*n *=* *35) and one milk sample spiked with FMDV A/TAN/01/2013 (1:20 v/v) were tested in parallel with the Enigma ML^®^ and the standard rRT‐qPCR at the FLI and TPI for the milk sample (Table 1). In addition, cattle epithelial suspensions prepared from field outbreak samples (*n* = 53), two positive control samples (tongue epithelial suspensions prepared from SAT1 and O naturally infected cattle) and two negative epithelial samples were tested with both systems at the SUA in East Africa.

**Table 1:** Diagnostic performance of the Enigma ML^®^ on archival clinical samples in a European laboratory

Virus	Type	Origin	Enigma ML^®^ C_T_	Enigma ML^®^ Printout	rRT‐qPCR
A_22_ Iraq	Epithelium 5 dpi	Bovine	22	POS	20.14
	Epithelium 7 dpi	Porcine	24	POS	20.96
	Epithelium 21 dpi	Ovine	33	POS	30.73
	Saliva 3 dpi	Bovine	27	POS	24.64
	Saliva 5 dpi	Bovine	31	POS	29.25
	Saliva 12 dpi	Bovine	***34***	***NEG***	***37.58***
	Saliva 3 dpi	Porcine	34	POS	32.49
	Saliva 5 dpi	Porcine	28	POS	27.15
	Saliva 3 dpi	Ovine	***35***	***NEG***	***36.82***
	Saliva 5 dpi	Ovine	35	POS	35.15
	Saliva 12 dpi	Ovine	No C_T_	NEG	38.21
O_1_ Manisa	Epithelium 5 dpi	Bovine	30	POS	26.89
	Epithelium 3 dpi	Porcine	21	POS	17.6
	Epithelium 5 dpi	Ovine	21	POS	17.02
	Saliva 3 dpi	Bovine	23	POS	17.94
	Saliva 5 dpi	Bovine	***32***	***NEG***	***32.6***
	Saliva 12 dpi	Bovine	No C_T_	NEG	37.67
	Saliva 3 dpi	Porcine	***33***	***NEG***	***28.35***
	Saliva 5 dpi	Porcine	***33***	***NEG***	***28.42***
	Saliva 11 dpi	Porcine	No C_T_	NEG	36.66
	Saliva 3 dpi	Ovine	***31***	***NEG***	***28.86***
	Saliva 5 dpi	Ovine	27	POS	25.58
	Saliva 12 dpi	Ovine	No C_T_	NEG	No C_T_
Asia 1 Shamir	Epithelium 4 dpi	Bovine	23	POS	18.37
	Epithelium 8 dpi	Porcine	31	POS	27.08
	Saliva 3 dpi	Bovine	***32***	***NEG***	***32.72***
	Saliva 5 dpi	Bovine	20	POS	24.59
	Saliva 12 dpi	Bovine	No C_T_	NEG	No C_T_
	Saliva 3 dpi	Porcine	No C_T_	NEG	36.41
	Saliva 5 dpi	Porcine	32	POS	32.79
	Saliva 3 dpi	Ovine	No C_T_	NEG	No C_T_
	Saliva 5 dpi	Ovine	No C_T_	NEG	36.21
	Saliva 12 dpi	Ovine	No C_T_	NEG	No C_T_
A/TAN/01/2013	1:20 Spiked Milk	Bovine	25	POS	22.36
Negative controls	Saliva	Bovine	No C_T_	NEG	No C_T_
	Saliva	Porcine	No C_T_	NEG	No C_T_
	Saliva	Ovine	No C_T_	NEG	No C_T_
	Milk	Bovine	No C_T_	NEG	No C_T_

In bold and italics: Samples called as negative on the Enigma ML^®^ printout that were corrected to positive after raw data investigation. C_T_, Cycle threshold; DPI, days post‐infection; POS, positive; NEG, negative.

### 2.5 European inter‐laboratory proficiency test

The performance of the Enigma ML^®^ was further evaluated in a collaboration of four European laboratories: FLI, TPI, CODA‐CERVA‐VAR (Veterinary and Agrochemical Research Centre, Belgium) and SVA (National Veterinary Institute, Uppsala, Sweden). A sample panel was provided by TPI, blinded and sent to the participating laboratories. The sample panel consisted of 12 samples (Table 2): Nine FMDV‐positive samples (sample 1 to 9, representing serotypes O, A, Asia 1, SAT1 and SAT2), one differential diagnostic sample of swine vesicular disease virus (SVDV ITL 3/2007 RS4 4/4/07 1:50) and two negative samples (uninfected cell culture supernatants, sample 10 to 12). All samples were tested in duplicate.

**Table 2:** Performance of the Enigma ML^®^ in the European inter‐laboratory proficiency test

Sample ID	Sample Name	Expected result	Laboratory 1		Laboratory 2		Laboratory 3		Laboratory 4
1	FMDV O IRN/2006: RS1 1:8	+	POS	POS	POS	POS	POS	POS	POS
2	FMDV O IRN/2006 RS1 1:40	+	POS	POS	POS	POS	POS	POS	POS
3	FMDV A TUR 2/2006 BTY1 1:30	+	POS	POS	POS	POS	POS	POS	POS
4	FMDV A TUR 2/2006 BTY1 1:60	+	POS	POS	POS	POS	POS	POS	POS
5	FMDV O ETH 43/2006 RS2 1:40	+	POS	POS	POS	POS	POS	POS	POS
6	FMDV O ETH 43/2006 RS2 1:500	+	POS	POS	POS	POS	POS	POS	POS
7	FMDV Asia 1 HKN 1/2005 RS2 1:100	+	POS	POS	POS	POS	POS	POS	POS
8	FMDV SAT 1 BOT 14/2006 RS2 1:8	+	POS	POS	POS	POS	POS	POS	POS
9	FMDV SAT 2 BOT 8/2006 RS2 1:8	+	POS	POS	POS	POS	POS	POS	POS
10	SVDV ITL 3/2007 RS4 1:50	−	NEG	NEG	NEG	NEG	NEG	NEG	NEG
11	MEM	−	NEG	NEG	NEG	NEG	NEG	NEG	NEG
12	MEM	−	NEG	NEG	NEG	NEG	***POS***	NEG	NEG

Each laboratory (except laboratory 4) tested the samples in duplicate. POS: positive; NEG. Negative. SVDV: swine vesicular disease virus Laboratory 1: FLI, Laboratory 2: CODA‐CERVA, Laboratory 3: SVA, Laboratory 4: TPI. ***Bold;*** false positive. MEM, minimum essential media; BTY, bovine thyroid; RS: IB‐RS‐2 swine kidney cells.

### 2.6 Ethics statement

All clinical samples used in this project were samples collected by local authorities in endemic countries and submitted to TPI or SUA, or archival samples from experimental studies at FLI that had been reviewed by an independent ethics commission and approved by the competent authority (State Office for Agriculture, Food Safety and Fisheries Mecklenburg‐Vorpommern, Rostock, Germany).

## 3 RESULTS AND DISCUSSION

Rapid and reliable diagnostic systems can play an integral role in the detection, monitoring, control and subsequent eradication of animal diseases and pathologies such as FMD. Especially in non‐specialized, front‐line laboratories with only basic equipment, for example in rural Africa, a demand exists towards test systems that are simple to perform, robust, inexpensive, and that yield unambiguous results for easy reporting (Knight‐Jones et al., 2016). In the recent past, several capacity building projects have sought to provide basic laboratories all over the world with reliable diagnostic systems for notifiable animal diseases. During these projects, real‐time PCR machines and other technology have been transferred with initial training. However, unfortunately, the availability and expense of consumables within the donor countries have often been inhibitive to the sustainability of these initiatives. For this reason, reliable and easy to handle test systems without delicate technology are still needed. In this context, different candidate systems were explored within the EU‐funded RAPIDIA‐Field project (grant agreement no. 289364), among them a new automated molecular diagnostic platform, the Enigma ML^®^ instrument, which combines RNA extraction, FMDV rRT‐qPCR and a sample reporting pipeline suitable for deployment into simple field laboratories. Its evaluation in different laboratories and within an inter‐laboratory comparison test is reported here.

**Figure 1:**
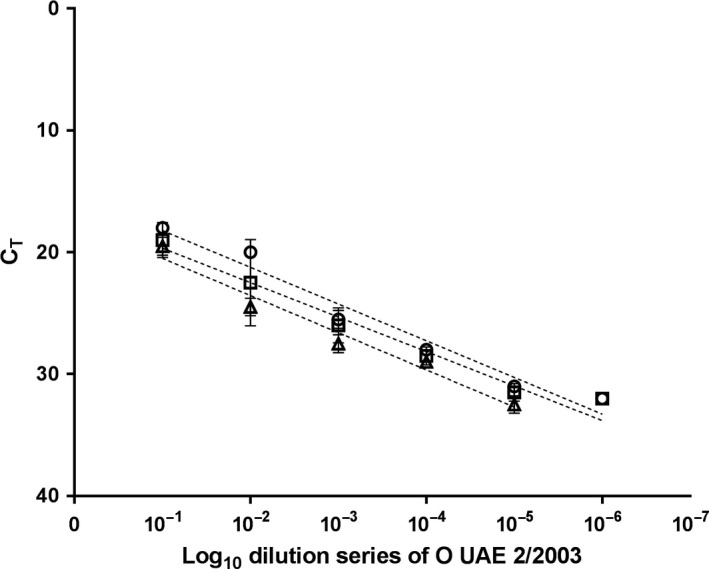
Analytical sensitivity of Enigma ML ^®^ in comparison with the gold standard rRT‐qPCR using FMDV‐spiked epithelium as the sample matrix. Circle: MagNa Pure LC ^a^ and Mx3005P^b^; Square: Enigma ML ^®a^ and Mx3005P^b^; Triangle: Enigma ML ^®a^ and ^b^; C_T_: Cycle threshold; ^a^extraction platform; ^b^real‐time RT‐PCR platform (rRT‐qPCR). O UAE 2/2003 baby hamster kidney (BHK‐21) passage 1

Initial tests were carried out to determine the limit of detection of the Enigma ML^®^ compared to a standard RNA extraction and rRT‐qPCR. To this means, a 10‐fold dilution series of FMDV O/UAE 2/2003 was used (Figure 1). When testing RNA extracted with the MagNA Pure LC robot in the rRT‐qPCR on the Mx3005P, the last positive dilution, that is the limit of detection, was a 10^−6^ dilution of the original material. RNA extracted by the Enigma ML^®^‐tested positive to 10^−5^ dilution. This one‐log reduction in analytical sensitivity observed when the RNA was extracted on the Enigma ML^®^ and the rRT‐qPCR was performed on the Mx3000P, and was also observed when both steps were performed on the Enigma ML^®^. Previous studies have shown that the analytical sensitivity of rRT‐qPCR assays is maintained when lyophilized reagents are used (Howson et al., 2015). Therefore, it is likely that the extraction protocol on the Enigma ML^®^ is less efficient than a standard laboratory extraction robot, but the reason for the reduced efficiency and possible remedies is unclear. It has to be noted that the limit of detection was not fully quantified in terms of genome copies, and that differences between the methods mostly occurred in dilution steps that were only weakly positive in the standard method (C_T_ –values > 30). Nevertheless, samples of clinically diseased animals contain higher genome loads (Murphy, Bashiruddin, Quan, Zhang, & Alexandersen, 2010; Pacheco, Stenfeldt, Rodriguez, & Arzt, 2016), and thus, the apparent lower sensitivity of the Engima ML^®^ platform is unlikely to affect the ability of this platform to reliably confirm FMDV infection in clinical animals.

For the clinical samples analysed in a European laboratory, mean C_T_ values obtained from duplicate testing with the routine diagnostic rRT‐qPCR ranged from 17.0 (ovine vesicle, 5 dpi, O_1_ Manisa) to 38.2 (ovine saliva, 12 dpi, A_22_ Iraq) (Table 1). The C_T_ values obtained from the raw data generated by the Enigma ML^®^ ranged from 20 (bovine saliva, 5 dpi, Asia 1 Shamir) to 35 (two ovine saliva samples, 3 dpi and 5 dpi, A_22_ Iraq) (Table 1). Using standard methods, 30 samples were positive and eight negative (no C_T_) (Table 1). The Enigma ML^®^ raw data revealed 25 positive samples and 13 negative samples, whereby four of these negative tested samples had high C_T_ values ranging from 36.2 to 38.2 in the routine rRT‐qPCR (Table 1). One sample that tested positive with a C_T_ of 34 on the Enigma ML^®^ had a C_T_ value of 37.6 in routine rRT‐qPCR. The printout stated 20‐positive samples and eight‐negative samples of which five samples were corrected to positive after the Enigma ML^®^ raw data were evaluated by an expert. At present, this raw data evaluation is not possible for the end‐user, and it is ultimately not desirable to have to rely on expert knowledge for the data analysis. In this respect, going forward the software of the Enigma ML^®^ may need improvement to give expert users access to background data and amplification curves, but at the same time, it improves the automated result calling for less skilled operators.

**Figure 2:**
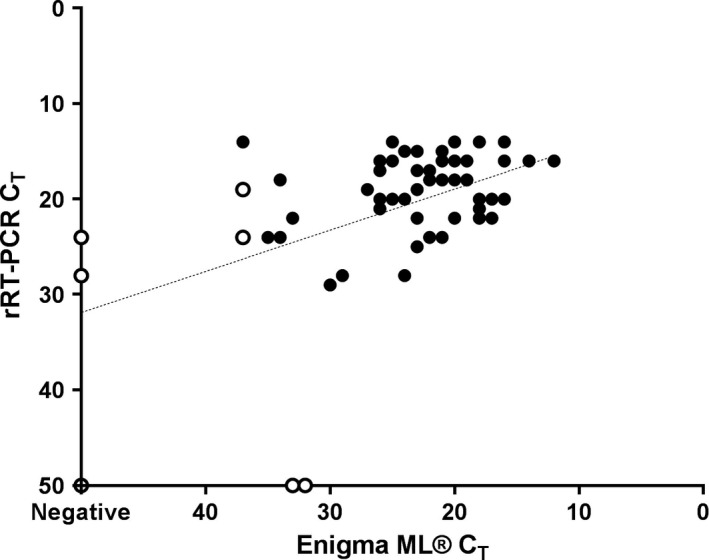
Diagnostic performance of the Enigma ML ^®^ compared to standard rRT‐qPCR using field samples of cattle epithelium in an African laboratory. Open circles were reported as negative on the Enigma ML ^®^ printout; closed circles were reported as positive on the Enigma ML ^®^ printout

Validation of the performance of the Enigma ML^®^ in an African laboratory setting was carried out at SUA. For this validation, 53 field epithelial samples collected from cattle displaying vesicular lesions (*n* = 51 FMDV positive, *n* = 2 FMDV negative) along with two positive (tongue epithelial suspensions from FMDV SAT1 and O naturally infected cattle) and two negative controls (tongue epithelial suspensions healthy cattle) were tested on the Enigma ML^®^ and compared to the standard laboratory RNA extraction and rRT‐qPCR pipeline used at SUA (Figure 2). There was 100% concordance between the Enigma ML^®^ and the standard rRT‐qPCR for the four control samples. The Enigma ML^®^ also correctly classified 48 of the 53 epithelial samples (90.6%), but five samples which were considered positive by standard rRT‐qPCR were reported as negative by the Enigma ML^®^ printout. These samples when assayed using the gold standard qRT‐PCR had an average C_T_ value of 24, and therefore, training the algorithms of the Enigma ML^®^ to recognize these samples as positive is required for future improvement of the system to ensure reliability of automated text only result calling.

When the proficiency test panel was analysed in four European laboratories, the relative sensitivity was 100% with all positive samples being accurately reported by the Enigma ML^®^ in all laboratories (Table 2). One participant reported a positive result in one duplicate for the negative sample 12, resulting in a relative specificity of 95% (Table 2). Based on all aliquots tested, the overall accuracy was 99%, and 83 of 84 aliquots were correctly identified across four laboratories, underscoring the robustness of the Enigma ML^®^ platform.

Overall, the Enigma ML^®^ displayed good concordance when compared to the standard laboratory RNA extraction and RT‐qPCR pipeline for clinical and laboratory‐prepared samples. Provided that future validation and optimization of the RNA extraction step can bring its performance to match that of standard laboratory extraction robots, the comparative data for the lyophilized reagents indicate that it is possible to generate a stabilized assay with equivalent (or better) performance than the wet‐assay format.

The data presented here show that it is possible for a fully automated molecular diagnostic platform, which is easy to use and requires minimum input from the operator, to rapidly detect FMDV in a range of clinical samples within 90 min. The data here can be therefore also used as a model for any other point‐of‐care assay using molecular detection techniques. Furthermore, our data open the possibility for deployment of this, or similar, sensitive molecular platforms into simple field laboratories within either endemic or exotic disease settings. Preliminary evaluation of the potential of milk as a diagnostic sample for detection of FMDV using the Enigma ML^®^ demonstrated promise, with the reporting of positive results for the spiked sample and negative results obtained from unspiked milk.

The Enigma ML^®^ platform is ideally suited for resource‐limited settings. Procurement and storage of consumables are simplified by the use of fully contained single‐use cartridges with lyophilized reagents. The minimal requirements for user intervention in the testing and reporting of results allow unskilled staff to use highly sensitive and specific molecular diagnostic tools successfully. Future validation to include a greater sample data set would increase confidence in the test, and commercialization of this particular assay, as well as other tests that might also exploit this format, will depend upon demand and interest from customers.

## CONFLICT OF INTEREST

James Wood and Paul Martin were employees of Enigma Diagnostics and the reported work was partially funded by the RAPIDIA‐Field project . All laboratory work and evaluation of the equipment were undertaken by staff from TPI, FLI, CODA‐CERVA‐VAR, SVA and SUA. Yves Van der Stede is currently employed with the European Food Safety Authority (EFSA) in the BIOCONTAM Unit that provides scientific and administrative support to EFSA's scientific activities in the area of Microbial Risk Assessment. The positions and opinions presented in this article are those of the authors alone and are not intended to represent the views or scientific works of EFSA.
